# Risk Perception and COVID-19

**DOI:** 10.3390/ijerph17093114

**Published:** 2020-04-29

**Authors:** Liliana Cori, Fabrizio Bianchi, Ennio Cadum, Carmen Anthonj

**Affiliations:** 1 (CNR-IFC) Research Unit of Environmental Epidemiology and Disease Registries, Institute of Clinical Physiology, National Research Council, 56124 Pisa, Italy; fabrizio.bianchi@ifc.cnr.it; 2 Department of Hygiene and Health Prevention and Complex Operative Unit Environmental Health and Innovative Projects, Health Protection Agency, 27100 Pavia, Italy; ennio_cadum@ats-pavia.it; 3 The Water Institute, Department of Environmental Sciences and Engineering, Gillings School of Global Public Health, University of North Carolina at Chapel Hill, Chapel Hill, NC 27516, USA; carmen.anthonj@email.unc.edu

**Keywords:** risk perception, environmental health, decision making, risk governance, risk communication, stakeholder participation, COVID-19

## Abstract

The ongoing COVID-19 pandemic is shaking the foundations of public health governance all over the world. Researchers are challenged by informing and supporting authorities on acquired knowledge and practical implications. This Editorial applies established theories of risk perception research to COVID-19 pandemic, and reflects on the role of risk perceptions in these unprecedented times, and specifically in the framework of the *International Journal of Environmental Research and Public Health* Special Issue “Research about risk perception in the Environmental Health domain”.

## 1. Introduction

The call for articles for the *International Journal of Environmental Research and Public Health* Special Issue “Research about risk perception in the Environmental Health domain” was proposed at the beginning of 2020 as part of multidisciplinary efforts to understand the complex interactions between people and the environment.

Oftentimes research on environment and health sheds lights on the fact that the prevention or limitation of risks to people and biodiversity often arises in contexts that are conflictual from a social and scientific point of view, especially when anthropogenic pressures are at stake. We had the ambition of observing ongoing improvements in risk perception studies. We included the different perspectives of policymakers, citizens, and stakeholders to reinforce, on the one hand, the validity of the research results, and, on the other, the usability of the results in decision making all along the governance cycle. The study of risk perception has, in fact, become increasingly relevant with the recognition that beliefs, knowledge, values, and attitudes influence not only decisions but also behaviors and, directly, the exposure of people to environmental pressures.

However, the ongoing COVID-19 pandemic modified the whole picture around us, and is shaking the foundations of public health governance all over the world. As the weeks go by, the impossibility of imagining the scope of the consequences we will face in the near future is emerging. This notwithstanding, researchers are challenged by informing and supporting authorities with acquired knowledge and practical implications. What seems possible today is to try to understand public reactions, applying established theories of risk perception research to COVID-19 and reflecting on the utilization of this knowledge to improve health risk communication, build trust, and contribute to a collaborating governance.

The current interdisciplinary research and reviews on risk perception in the environment and the health domain will form the core of the Special Issue in progress, but the challenge posed by the pandemic is a proper emerging issue that must be tackled and examined.

The connections between the environment and health issues must be explained, as there is in fact a substantial difference between infectious diseases such as COVID-19 and non-communicable diseases caused by the impact of pollution on human health. Infectious diseases have a single necessary cause, although sometimes not sufficient, and spread with deterministic mechanisms, while non-communicable ones have multiple causes which are usually neither necessary nor sufficient and act through a “causation network”. If the distal causes of the emergence of the novel SARS-CoV-2 are to be found in climate change, ecosystem changes, or migrations, then the main causes that influence the COVID-19 epidemic are all the factors that facilitate contagion. These factors include the presence of outbreaks among people travelling, contacting each other, as well as many social, cultural, psychological, and environmental cofactors that play an important role directly or as effect modifiers.

## 2. The Pandemic

Within only three months, the COVID-19 pandemic has become the most severe global health challenge since the Spanish Flu one century ago [1]. The novel coronavirus has already reached 213 countries, areas or territories with cases around the world, with over 3 million confirmed cases and more than 200,000 deaths (status quo on April 28, 6:00 CET) [2]. While some governments have been implementing drastic measures to slow down the spread, such as severe travel restrictions, the closing of borders, lockdowns, curfews, and the limitation of personal contact to family plus one non-family member only, others have decided to take softer actions or mainly voluntary measures.

Along with the pandemic, fear spreads and grows. This is not a novel phenomenon. The reason is traceable in risk communication theory, found in the history of public health, and confirmed also by the current developments of COVID-19.

By now, the majority of people around the world have heard of the coronavirus and of the need to practice hand hygiene and social distancing to prevent its spread. But while some individuals strictly adhere to the restrictions, others ignore or delay the governments’ rules and mingle in crowds in public places, at beaches, or in their homes. The fact that individuals in these times of joint challenge act so differently indicates that the risk perception relating to this novel virus strongly differs between different places and individuals. This means, moreover, that risk perception is potentially a strong modifier of the epidemic evolution, since it can influence the number of new positive cases.

The coronavirus outbreak was declared a public health emergency of international concern by the World Health Organization on 30 January 2020, and since then, many governments have declared a state of emergency—allowing the reallocation of resources and for drastic measures to be taken. War metaphors are commonly used by many governments and media; the global community and national and local governments “fight against” and hope to “win the war against the pandemic” [3,4]. A U.S. Navy hospital ship with a 1000 bed capacity was deployed in New York City, the new epicenter of COVID-19, as a response to the pandemic [5]. This makes this metaphor come to life—and increases the fear further.

In the multitude of discussions and articles on different media, we find it’s possible to find all the typical ingredients of a time of epidemics: accusations, plots, exploitation, obscure interests, misinformation, and misbelief. There are a few that try to reassure too much and many that demand collaboration and rationality in facing the complex scenarios (that no one can understand in an exhaustive way).

Never-forgotten anxieties re-emerge from the distant (plague) and closer (smallpox) past when a novel disease rapidly appears, crossing borders and approaching worlds that seemed very far away.

## 3. Risk Perception during the COVID-19 Epidemic

One by one, the elements that characterize the perception of risk emerge, and should be considered if the management of risk communication by governments is to be based on rationality and consciousness.

Risk perceptions refer to people’s intuitive evaluations of hazards that they are or might be exposed to [6], including a multitude of undesirable effects that people associate with a specific cause [7]. Risk perceptions are interpretations of the world. The evaluation of risks is influenced by numerous individual and societal factors, and different social, cultural, and contextual factors influence risk perception. These go beyond the classic hazard attributes and are based on experiences, beliefs, attitudes, judgments, (mis)conceptions, and feelings, as well as wider social, cultural, and institutional processes [8]. Although risk perceptions act as triggers for precautionary action [9], the engagement in preventive health behaviors is not merely determined by the awareness of objective health risks, but also influenced by health beliefs and specific health cognitions [10].

The results of an extensive set of research has allowed us to highlight some key features explaining the perception of risk and its influence on decision-making: familiarity, controllability, voluntary exposure, potential catastrophic, equity, the immediacy of danger, and the level of knowledge [11]. According to this approach, the perception of risk is the subjective judgment that people create regarding the characteristics, severity, and way in which the risk is managed. One of the key elements is the sense of outrage and indignation that the risk produces, which multiplies anxiety and quickly runs through society. In fact, Sandman et al. [12] propose a definition of risk as a product between hazard and outrage. The risk is intended as a probabilistic assessment, produced comparing the probability that an event occurs and the seriousness of the potential damage. It is largely influenced by outrage, which constitutes then a key element in capturing (or understanding) the estimated risk, which concerns both the nature of the risk and its management.

The elements that increase or mitigate fear and risk perceptions have been extensively examined and discussed [13].

▪ **Voluntariness**: If the risk is taken voluntarily, it seems to be perceived as lower. This is applicable to smoking, driving fast cars, and practicing dangerous sports. If the risk is imposed by others (external forces) or is uncontrollable it is perceived as greater. The risk of COVID-19, like of all epidemics, is not only involuntary but clearly uncontrollable by individuals, and difficult to control even by health authorities and governments.
▪ **Knowledge**: An unusual risk is perceived as more frightening, and the novel coronavirus circulating in these times has been presented as a completely unknown virus, with testing that had to be newly developed to detect it and without a remedy to cure it. A risk of natural origin provokes less fear than one caused by a human, and conspiracy theories that accompany the novel threat increase the feeling of discomfort and fear. A reversible risk is perceived as associated with less anxiety as compared to an irreversible risk. A risk that comes with benefits could also be acceptable, as is the case for technologies/industries that create jobs or provide services while impacting social justice, where social decisions need to be balanced against different needs and values. In the case of the novel coronavirus, we can see how individuals, communities, and countries suffer from disadvantages. The fear of death hangs over all the infected—regardless of the lethality rate.
▪ **Visibility**: an invisible risk factor is perceived as more hazardous than a visible one (e.g., a chemical plant, an incinerator, or radio base stations).
▪ **Trust**: If there is any confidence in those managing the risk at all, it is at present not perceived as high. In the case of COVID-19, many individuals raise their voices, even in an opportunistic way, to undermine the credibility of health institutions. Once trust is lost, it is very difficult to regain. Divergences within the scientific community in an emergency situation can have devastating effects if a consensus is not produced. Public authorities need to pay particular attention to sharing knowledge, finding alliances in society, and building confidence—which, in turn, would reduce the fear.

Fear is inherent in the COVID-19 characteristics and is not completely manageable, especially with generic calls to dominate fear, and an excess of public concern around the difficult management of such a complex problem cannot be avoided.

Thinking ahead and about the future, our current fear and perception of risk can serve as a useful tool to promote increased preparedness and improved response by the health sector. Moreover, on a larger scale, fear can promote consciousness and care towards the planet, following the philosopher Hans Jonas’ declaration that an authentic responsibility towards the future implies an “heuristic of fear”. Fear that is rooted in our biological baggage can be used as a tool that drives us to prudence. In order to ensure the inviolable obligation to inform and the right to know, conventional and social media offer direct updates on the number of infected people and each new death, accompanied by the number of citizens subjected to containment measures (this number grows and rapidly reaches the whole population). Each death takes on enormous weight and increases fear and bewilderment.

In addition, the World Health Organization (WHO) issues daily reports with the necessary level of detail to assess not only the overall extent of the crisis but also its progression. The reports show the resident population, the number of confirmed cases per day and the cumulated number of cases, the number of deaths per day and the cumulative lethality per day, and other information, including suspected cases and the trend curves of the epidemic [14]. The calculable lethality rate, using not sick cases but all infected cases as a denominator (which, fortunately, are or will become partially ill), could provide important information on the different time periods if the rate is true and not underestimated, as it is reasonably likely to be since not all positive cases are recorded. The increase in general mortality and the admissions to hospitals in high intensity of care unit would be more appropriate for understanding the dynamic of the epidemic.

Using the case fatality rate (CFR) as a proxy, in Italy, at the high point of the epidemic, the CFR was over 12.3%, slightly higher than in Spain (9.7%) and France (8.6%) but much higher than that registered in the U.S. (2.8%) and most other countries. Beyond the in-depth analysis of the causes, the absolute number of deaths creates a great concern, especially when those deaths are concentrated in a limited geographical space and in confined places, such as nursing homes, residences for the elderly, or hospitals [15].

There is a macabre juggle with information that is not found in any other field of health. Seasonal flu epidemics cause more than 10 times the number of infections of coronavirus but have a lethality that is 100 times lower, partly because of the high immunization coverage.

Repetitive and expected events usually do not arouse fear but, if anything, apprehension.

More concern arises when the causes are not explained.

In the case of COVID-19, a good spatial–temporal contextualization is needed to support the definition of scenarios for the post-epidemic phase. A “stop and go” strategy, as envisaged by Imperial College London, seems possible, particularly if we consider the need for flexible measures and limited knowledge of both the duration of the infectious capacity and identification of acquired immunity [16]. Related parameters would be useful in guiding decision-makers in their choices [17].

The role of the factors identified above—voluntariness, knowledge, and trust—appears crucial in planning communication actions suitable for the next delicate phase. In order to communicate strategies we need to evaluate the application and effectiveness of different protective measures for different age groups and individuals with different health conditions. The active involvement of communities—which are showing extraordinary creative, mobilization, and support skills—can transform the sense of outrage into an assumption of personal and collective responsibility. Moreover knowledge entails the growth of collective awareness, the increase in self-efficacy, and the empowerment in contributing to political decision-making. Mutual trust and the reliance on local communication networks between peers could exponentially increase the possibilities of applying flexible measures. Attention to the aspects of equity and the respect for rights and privacy should be maximized, and the war metaphors should be shifted towards health- and wellbeing-promoting concepts linked to healing, collaboration, and solidarity [18].

## References

[1] Pederson T. (2018). Where Are We, a Century After the “Spanish Flu”?. Faseb J..

[2] World Health Organization Coronavirus Disease (COVID-19) Pandemic. https://www.covid19.who.int.

[3] Levenson E. Officials Keep Calling the Coronavirus Pandemic a ‘War’. Here’s Why. CNN, USA. https://www.cnn.com/2020/04/01/us/war-on-coronavirus-attack/index.html.

[4] Gebrekidan S. The World Has a Plan to Fight Coronavirus. Most Countries Are Not Using it. https://www.nytimes.com/2020/03/12/world/coronavirus-world-health-organization.html.

[5] Cooper H., Gibbons-Neff T. Navy Hospital Ship Reaches New York. But It’s Not Made to Contain Coronavirus. https://www.nytimes.com/2020/03/30/us/politics/coronavirus-comfort-hospital-ship-new-york.html.

[6] Rohrmann B. Risk Perception, Risk Attitude, Risk Communication, Risk Management: A Conceptual Appraisal. Proceedings of the International Emergency Management Society Annual Conference.

[7] Rohrmann B., Renn O., Renn O., Rohrmann B. (2000). Risk perception research. An introduction. Cross-cultural Risk Perception. A Survey of Empirical Studies.

[8] Pidgeon N. (1998). Risk assessment, risk values and the social science programme: Why we do need risk perception research. Reliab. Eng. Syst. Saf..

[9] Wiedemann P.M., Schütz H. (2005). The precautionary principle and risk perception: Experimental studies in the EMF area. Env. Health Perspect..

[10] Renner B., Schupp H., Vollmann M., Hartung F.-M., Schmälzle R., Panzer M. (2008). Risk perception, risk communication and health behavior change. Z. Für Gesundh..

[11] Cerase A. (2017). Risk and Communication. Theories, Models, Problems.

[12] Sandman R., Weinstein N.E., Hallman W.K. (1998). Communications to reduce risk underestimation and overestimation. Risk Decis. Policy.

[13] Slovic P., Krimsky S., Golding D. (1992). Perception of risk: Reflections on the psychometric paradigm. Social Theories of Risk.

[14] World Health Organization Coronavirus Disease (COVID-19) Situation Reports. https://www.who.int/emergencies/diseases/novel-coronavirus-2019/situation-reports.

[15] Johns Hopkins University of Medicine COVID-19 Dashboard by the Center for Systems Science and Engineering (CSSE) at johns Hopkins University (JHU). https://coronavirus.jhu.edu/map.html/.

[16] Ferguson N., Laydon D., Nedjati Gilani G., Imai N., Ainslie K., Baguelin M., Bhatia S., Boonyasiri A., Cucunuba Perez Z.U., Cuomo-Dannenburg G. 2020. Report 9-Impact of Non-Pharmaceutical Interventions (NPIs) to Reduce COVID-19 Mortality and Healthcare Demand. https://www.imperial.ac.uk/mrc-global-infectious-disease-analysis/covid-19/report-9-impact-of-npis-on-covid-19/.

[17] Flaxman S., Mishra S., Gandy A., Unwin H., Coupland H., Mellan T., Zhu H., Berah T., Eaton J., Perez Guzman P. Report 13-Estimating the Number of Infections and the Impact of Non-Pharmaceutical Interventions on COVID-19 in 11 European Countries. https://www.imperial.ac.uk/mrc-global-infectious-disease-analysis/covid-19/report-13-europe-npi-impact/.

[18] Nie J.B., Gilbertson A., De Roubaix M., Staunton C., Van Niekerk A., Tucker J.D., Rennie S. (2016). Healing Without Waging War: Beyond Military Metaphors in Medicine and HIV Cure Research. Am. J. Bioeth..

